# Hsa-miR-31 Governs T-Cell Homeostasis in HIV Protection *via* IFN-γ-Stat1-T-Bet Axis

**DOI:** 10.3389/fimmu.2021.771279

**Published:** 2021-11-05

**Authors:** Lingyan Zhu, Chao Qiu, Lili Dai, Linxia Zhang, Meiqi Feng, Yu Yang, Chenli Qiu, Anli Zhang, Jun Huang, Ying Wang, Ying Wan, Chen Zhao, Hao Wu, Jianxin Lyu, Xiaoyan Zhang, Jianqing Xu

**Affiliations:** ^1^Shanghai Public Health Clinical Center and Institutes of Biomedical Sciences, Fudan University, Shanghai, China; ^2^School of Laboratory Medicine and Life Science, Wenzhou Medical University, Wenzhou, China; ^3^Beijing You’an Hospital, Capital Medical University, Beijing, China; ^4^Department of AIDS/STD, Shanghai Municipal Center for Disease Control and Prevention, Shanghai, China; ^5^Biomedical Analysis Center, Army Medical University, Chongqing, China

**Keywords:** miR-31, HIV-1, disease progression, CD4+ T cell, T-bet, STAT1

## Abstract

It remains poorly defined whether any human miRNAs play protective roles during HIV infection. Here, focusing on a unique cohort of HIV-infected former blood donors, we identified miR-31 (hsa-miR-31) by comparative miRNA profiling as the only miRNA inversely correlating with disease progression. We further validated this association in two prospective cohort studies. Despite conservation during evolution, hsa-miR-31, unlike its mouse counterpart (mmu-miR-31), was downregulated in human T cell upon activation. Our *ex vivo* studies showed that inhibiting miR-31 in naïve CD4+ T cells promoted a transcriptional profile with activation signature. Consistent with this skewing effect, miR-31 inhibition led to remarkably increased susceptibility to HIV infection. The suppressive nature of miR-31 in CD4+ T cell activation was pinpointed to its ability to decrease T-bet, the key molecule governing IFN-γ production and activation of CD4+ T cells, by directly targeting the upstream STAT1 transcriptional factor for downregulation, thus blunting Th1 response. Our results implicated miR-31 as a useful biomarker for tracking HIV disease progression and, by demonstrating its importance in tuning the activation of CD4+ T cells, suggested that miR-31 may play critical roles in other physiological contexts where the CD4+ T cell homeostasis needs to be deliberately controlled.

## Introduction

HIV-1/AIDS has become a major infectious disease posing severe threats to human life and global economy. According to UNAIDS, the cumulative number of patients acquiring HIV/AIDS is estimated to be 77.5 million, half of whom were still alive at the end of 2020 (1). With differentiated CD4+ T cells as its primary target, HIV infection progressively deteriorated human immune system through three major phases: primary, chronic, and AIDS. The primary phase encompasses the first six months after virus infection, featuring rapid virus replication and consequently loss of CD4+ T cells due to activation of cell death pathways. The ensuing chronic phase is contrastingly marked by a decrease in viral replication alongside a generalized activation of immune system, a period that last variably with an average of 8-10 years (2). AIDS represents the final stage of HIV disease when patients display clinical manifestations including low CD4+ T cell counts and compromised cellular immunity that renders increased susceptibility to infection and tumor formation (3).

The recently increased survival of HIV patients largely attributes to the success of antiretroviral therapies (ART), which has transformed HIV from a terminal to a chronic disease (4). However, the eradication of AIDS requires a further exploration of biology underlying HIV infection, particularly the host factor determinants of virus biogenesis and transmission. MicroRNAs (miRNAs) are among the host factor determinants that researchers recently searched for correlation with progression of HIV/AIDS. Extensive studies have revealed a plethora of roles that miRNAs play in various biological processes, with important implications in disease pathogenesis and progression (5–7). The potential engagement of miRNAs has been also explored for HIV infection by several approaches. One approach attempted was to identify miRNA upregulated in activated CD4+ T cells as opposed to quiescent CD4+ T cells, which stems from that activation of CD4+ T cells is generally regarded as a prerequisite for effective HIV infection (8). The other related approach involved identifying miRNA whose expression is affected by HIV infection, based on the speculation that host can deploy miRNA to downregulate viral accessory genes (9) or host factors critical for viral replication (10–12), or vice versa, virus is smart enough to upregulate miRNA negatively regulating host restriction factor to facilitate its replication (13–15). There were also studies using bioinformatics to analyze the HIV genome to screen for potential target sites of known miRNAs (16, 17). Although a handful of miRNAs were thus identified, whether they truly play roles in HIV infection remain controversial as their functions have been most functionally examined in cultured cells without evidence demonstrating an association with clinical outcomes. An alternative, but more physiologically relevant, approach is determining the existence of miRNA whose expression is correlated with disease progression by analyzing blood samples from HIV patients with different clinical phenotypes. Most of HIV-infected patients experience a rapid decline in CD4 counts and accelerated development of AIDS. However, a small portion of them show a slow disease progression and can be further classified into several distinct groups according to immunologic or virologic parameters. Long-term non-progressor (LTNP) is generally defined as individuals who have been displaying stable CD4 T-cell count for over 8-10 years after diagnosis. LNTP is thus an immunologic definition. In contrast, elite controller (EC) and viremic controller (VC) are virologic definitions, defined, respectively, as patients with undetectable plasma viral loads (generally <50 copies/mL) for extended periods and those with plasma viral loads of 50–2000 copies/mL. EC is a rare group, only accounting for 4-12% of LNTPs, according to reported cohort studies (18, 19). By comparing miRNA expression between viremic HIV patients and LNTPs or ECs patients, a number of HIV disease-related miRNAs were identified and some of them, as exemplified by MiR-31, were found to be correlated with key parameters of disease progression such as virus load and CD4+ T-cell count (20, 21). However, due to the scarcity of studies, the values of these miRNA candidates in tracking HIV disease progression have yet been firmly validated. More importantly, there is little mechanistic insight into how these miRNAs regulate the HIV infection of CD4+ T cells, which constitutes the essential step towards their exploration as new targets for anti-HIV therapy.

Here, we analyzed the difference between LNTP and viremic patients in miRNA expression profiles, focusing on a unique cohort of HIV-infected former blood donors. Combining with following validation in the other patient cohort, we identified miR-31 (hsa-miR-31) as the only miRNA showing strong correlation with benign clinical consequences in both acute and chronic HIV infections. Importantly, we pinpointed a major protective mechanism of hsa-miR-31 against HIV infection, that is, hsa-miR-31 maintains the homeostasis of CD4+ T cells *via* directly targeting *STAT1* to tune the IFN-γ-Stat1-T-bet axis, a positive feedback loop known for governing the activation of CD4+ T cells. Together, our data not only established miR-31 as a reliable biomarker for tracking the HIV diseases progression, but also provided a mechanistic explanation shedding new light into basic T cell biology.

## Materials and Methods

### Study Subjects

The study subjects included individuals from 2 sources (1): chronically HIV-1 infected participants in Yun’cheng city, Shanxi province, China (2); newly infected MSM (men who have sex with men) in Beijing You’an hospital. Written informed consents were obtained from all participants. The overall study was reviewed and proved by the Ethics Committee of SHAPHC. HIV-infected Former blood donor cohort (FBD study): The 50 HIV-infected former blood donors donated plasma between 1995 and 1996 in three counties of Yuncheng city and were infected with HIV-1 from common-source of contaminated blood. The participants were screened and recruited at the beginning of 2009 (baseline of FBD study). The inclusion criteria were ART naïve subjects with no symptoms of AIDS or related opportunity illnesses. At the baseline, their ART-free statuses were confirmed by measuring the plasma concentration of antiretroviral drugs available in China. The loci of HLA-A, HLA-B, HLA-C and HLA-DR were also genotyped. For slow progressors, confirmative western blot analyses were conducted at two independent laboratories. After enrollment, participants attended quarterly visits unless subjected to antiretroviral medications. Absolute CD4^+^ T cell counts were measured at every visit and viral loads were assessed semi-annually. Immune activation was measured by the frequency of CD38 positive CD8^+^ T cells. The primary end point was defined as CD4^+^ T cell counts less than 350 cells/mL, initiation of long-term ART, AIDS or death (**Supplementary Table 1** for detailed information). MSM study: The 54 newly HIV-1 infected MSM were selected from a previous established cohort at Beijing You’an Hospital, in which HIV-1-negative high-risk MSM were screened every 2 months for recent HIV-1 infection, and had been followed up for 2 years after the first positive point. Plasma samples spanning seroconversion (from pre-infection to early chronic infection) were collected. The timing of estimated infection was determined by the history of risk exposure and laboratory measurements. Most of the samples reached the end point within 12 months.

**Cell lines**. HEK293T (embryonic kidney cell line, ATCC #CRL-3216) was cultured in DMEM (Corning #10-013-CV) supplemented with 10% FBS (BI #04-001-1acs) and 1% (Corning #30-002-CI).

### Taqman MicroRNA Array and Analysis of miRNA Array Data

PAXgene Blood miRNA Kit (QIAGEN, Hilden, Germany) was used for extraction and purification of total RNA, including miRNA, from whole blood stabilized in PAXgene Blood RNA Tubes (PreAnalytix, BD, UK). The resultant total RNA containing miRNA was stored at -80°C until analysis. Total RNA samples were reverse transcribed to cDNA using Megaplex RT Primers and the TaqMan MicroRNA Reverse Transcription kit (Applied Biosystens, Foster City, CA), followed by 12-cycle preamplification reactions using the Megaplex PreAmp Primers and TaqMan PreAmp Master Mix (Applied Biosystens, Foster City, CA). miRNA expressions were then quantified by real-time PCR with the TaqMan Universal PCR Master Mix and TaqMan Array Human MicroRNA A+B Cards v3.0 (Applied Biosystems, ABI) on an 7900HT (Applied Biosystems). Expression levels of 754 miRNAs in each sample were quantified, with U6 used as the internal control. Relative miRNA expression levels were expressed as -ΔCt values, calculated by subtracting Ct values of U6 from those of each miRNA, and used for analyses. Statistical and hierarchical cluster analyses of expression data were performed using Biometric Research Branch (BRB)-Array Tools version 4.1.0 Beta 2. miRNAs showing minimal variation or the percentage of missing value more than 20% across the set of arrays were excluded from the analysis, and those whose expression differed by at least 1.5-fold from the median in either direction in at least 20% of the arrays were retained. Hierarchical clustering analysis was performed with median-centered correlation and complete linkage. Significance Analysis of Microarrays (SAM), which give 95% confidence that the false discovery rate was less than 5%, was applied to search for the differentially expressed miRNAs that are correlated with CD4^+^ T cell counts and HIV RNA levels.

### Individual miRNA Quantification

The method for extracting total RNA from whole blood was described above. miRNA isolation from plasma was performed with miRNeasy RNA isolation kit (Qiagen). The RNA samples were reverse transcribed using stem-loop RT primer pools and TaqMan MicroRNA Reverse Transcription kit (Applied Biosystems), followed by pre-amplification PCR according to the manufacturer’s instruction. The resultant cDNA products were diluted and subsequently subjected to real-time qPCR assay. All the assays were performed in duplicate and small nuclear RNA, RNU6B, was used as the normalization control.

### RNA Analyses of Antagomir-Treated Cells

Total RNA was extracted from cells using a RNA isolation kit from Qiagen, and 500ng of purified total RNA was reverse transcribed using oligo (dT)-15mer primer and AMV Reverse Transcriptase (Promega) according to manufacturer’s protocol. The synthesized cDNA was subsequently analyzed by SYBR Green-based quantitative real-time PCR assay using a Promega kit and the following gene-specific primers: ACTB, forward primer, 5’- GCATGGGTCAGAAGGATTCCT-3’, revers primer, 5’- TCGTCCCAGTTGGTGACGAT-3’; T-bet, forward primer, 5’-CGGCTGCATATCGTTGAGGT-3’, revers primer, 5’-TAGGCAGTCACGGCAATGAA-3’; IFNG, forward primer, 5’-TGGCTTTTCAGCTCTGCATC-3’, revers primer, 5’- CCGCTACATCTGAATGACCTG-3’; STAT1, forward primer, 5’-CAGCTTGACTCAAAATTCCTGGA-3’, revers primer, 5’- TGAAGATTACGCTTGCTTTTCCT-3’;

### T-Cell Isolation

Primary peripheral blood mononuclear cells (PBMCs) were isolated from whole blood by Ficoll separation. The contained total CD4^+^ T cells or naïve CD4^+^ T cells were then isolated using human total CD4^+^ T cell isolation kit and human naïve CD4^+^ T cell isolation Kit (Stemcell), respectively. Cell purity was >95% as determined by flow cytometry with surface marker CD3+ CD4+ CD8- and CD3+ CD4+ CD8- CD45RA+ CCR7+ for total and naïve CD4+ T cells, respectively.

### Transfection of MicroRNA Inhibitors and Mimics and Activity Assay

Primary T cells were resuspended at 5× 10^6^ cells/mL in serum-free culture media X-VIVO15 (Lonza), 200µL of the resulting cell suspension was transferred to each well of a 96-well round-bottom microtiter plate. Transfection mixture was prepared by mixing 120 pmol of miR-31 antagomir (antagomiR-31) or control antagomir (antagoNC) and 0.6µL of TurboFect transfection reagent (Invitrogen) into X-VIVO15 to a final volume of 20µL, followed by incubation at room temperature for 20 min before adding to each well. Cells were then cultured in a humidified 37°C, 5% CO2 incubator for 48 hours before being collected for analysis. For the assessment of the effect of miR-31 on 3’ UTR of STAT1, 3’ UTR of STAT1 or its miR-31 site mutated version was subcloned into pMIR-REPORT firefly plasmid (Ambion) and co-transfected with a Renilla luciferase plasmid, and miR-31 mimics (Mimic-miR-31) or negative control microRNA (mimic-control) into HEK293T cells using TurboFect. The cells were collected at 24 hours post transfection for measurement of luciferase activities using the dual luciferase reporter assay system (Promega).

### RNA-Seq Analysis of Antagomir-Treated Naïve T Cells

Naïve CD4+ T cells isolated from PBMCs of healthy donors were transfected with antagomiR-31 or antagoNC as described above. Cells were collected 48 hours later in RNAzol (Molecular Research Center) and stored at -80°C until processed to RNA isolation using the Direct-zolTM RNA MiniPrep Kit (ZYMO). The RNA-seq library preparation, sequencing and analysis were performed by BioWavelet Ltd. (Chongqing, China). In brief, the library was constructed using EASY-RNAseq method (22) and then sequenced on Illumina HiSeq platform using the 150-bp pair-end configuration. Raw reads were parsed with Trimmomatic for adaptor and quality trimming before alignment to the human genome reference GRCh38.p7. Only reads assigned to a unique genomic locus were counted using FeatureCounts, and the resulting read counts were converted to FPKM values to measure gene expression. Gene set enrichment analysis of the expression data was performed using GSEA software (Broad Institute) as described (23); hierarchical cluster analysis was conducted based on average linkage and Euclidean distance metric using Multiexperiment Viewer (version 4.9, http://www.tm4.org) (24).

### Flow Cytometry and Cytometric Bead Array (CBA)

Flow cytometry was performed on a LSRFortessa Flow Cytometer (BD Biosciences) and the data were analyzed using FlowJo software. The following antibodies and regents were used: CD8 PB (clone RPA-T8) was from BD Biosciences; CD45RA PE-Cy7 (clone H2100), CCR7 APC (clone 3D12), CD38 PE-Cy7 (clone HIT2) were from eBioscience; CD3 PE/Dazzle594 (clone UCHT1), CD25 PE (clone BC96), HLA-DR ECD (clone L243), T-bet PE (clone 4B10) were from BioLegend; p24 FITC (clone KC57) was from Beckman; Live/Dead™ blue dye (Invitrogen) was used for distinguishing live and dead cells. Measurements of cytokines in cell culture medium were performed with BD™ Cytometric Bead Array Th1/2/17 kit according to the manufacturer’s protocol.

### HIV Infection *In Vitro*

Isolated CD4+ T cells were transfected with antagomiR-31 or antagomir-NC for 48 hours, followed by culturing in R10 medium (RPMI-1640 medium supplemented with 10% FBS) under stimulation with anti-CD3 and anti-CD28 for 3 days. The cells were subsequently washed and spin-infected (1200xg for 2 hrs at 25°C) with HIV-1 IIIB at 0.01 multiplicities of infection (MOI). After washing, cells were cultured in R10 supplemented with IL-2 (20 IU/ml) for 11 days, cells and supernatants were then collected for infectivity determination. Cells were stained with live/dead, CD3, and CD4 antibodies and after fixation and permeabilization, samples were stained with anti-p24 antibodies before submission to flow cytometry analysis. The concentrations of p24 in the supernatant were determined by ELISA (bioMerieux Industry) according to the manufacturer’s protocol.

### Western Blotting and IFN-γ Neutralization

Western blotting analyses were performed with whole cell lysates of antagomiR-31 or antgoNC treated naïve CD4+ T cells according to previously published procedures (25). Following antibodies were used: mouse anti-actin antibody (1:3,000; Cell Signaling Technology, #3700S), rabbit anti-STAT1 Ab (1:1,000; Cell Signaling Technology, #9172S), and rabbit anti-phospho-STAT1 Ab (Cell Signaling Technology, #9167S). The immunoblots was imaged and analyzed using Image Studio (LI-COR Biosciences). The anti-human IFN-γ antibody (clone B27) used for neutralization of IFN-γ in the cell culture medium and the IgG1κ isotype control antibody (Clone MG1-45, Cat. No. 401409) were purchased from BioLegend.

### ATAC-Seq

The service of ATAC-seq was provided by Active Motif Inc., using procedures as described previously (26). In brief, 200,000 cells in duplicates were used for transposition reaction. After purification with MinElute PCR purification kit (Qiagen), transposed DNA was amplified by PCR with a total of twenty-five cycles, which was determined as optimal by qPCR. Library was then size selected using AMPure XP beads to remove fragments greater than 800 bp or smaller than 100 bp. The eluted library was validated for size distribution by DNA ScreenTape Assay (Agilent, USA) and quantified with Qubit dsDNA High-sensitivity Assay Kit (Thermo Fisher Scientific) on a Qubit Fluorometer. Finally, all the samples were pooled and sequenced on a NextSeq 500 in a pair-end modality rendering 2 x 150bp sequences. After removing low-quality reads and duplicate reads, the ATAC-seq data were aligned to the human reference genome hg38 and the alignments were visualized on the UCSC genome browser.

### Statistical Analysis

All statistical analyses were performed using GraphPad Prism 7.0 (GraphPad Software). Survival curve differences were assessed using the log-rank-test. The Mann-Whitney U test was used to compare the difference between two groups, data from the same individuals were compared by the Wilcoxon matched-pairs t test. The degree of association between two variables was measured by Spearman correlation. In all cases, *P* values below 0.05 were considered statistically significant; those below 0.1 were considered to approach significance. All P values reported were 2-sided.

### Study Approval

The study was reviewed and approved by the Ethics Committee of Shanghai Public Health Clinical Center (SPHCC). Written informed consents were provided by all participants.

## Results

### miR-31 Predicts HIV -1 Disease Progression

**Figure 1:**
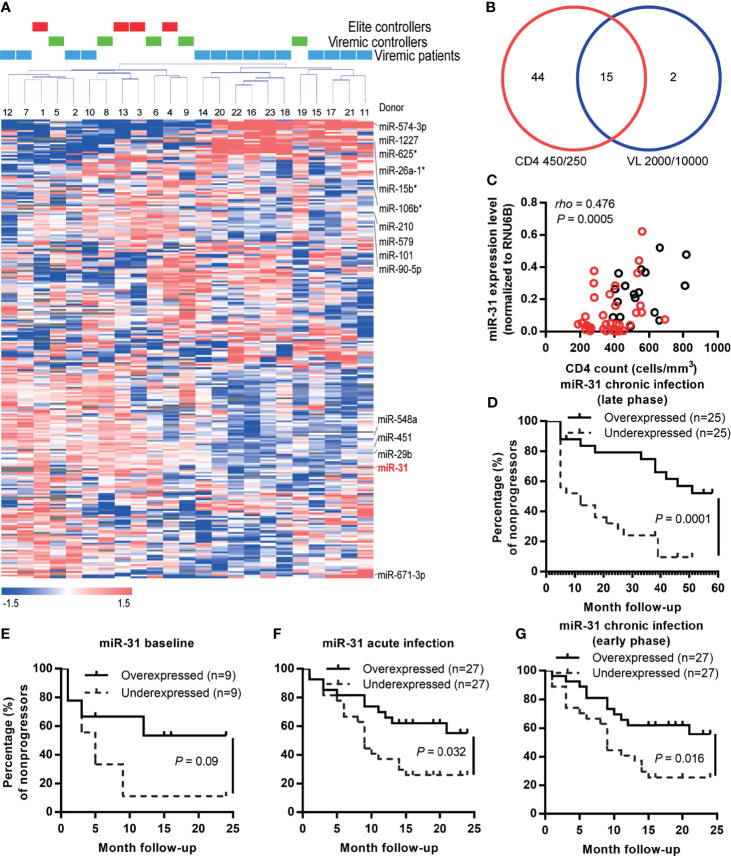
miR-31 is correlated with disease progression during both acute and chronic HIV-1 infection. **(A)** Unsupervised clustering of the 251 miRNAs. After normalization and filtering of the microarray data, 251 miRNAs were retained for further analysis. Average linkage hierarchical clustering was performed using a centered correlation metric. Twenty-three samples from the FBD study were clustered into 2 groups: the left cluster was a mixture of elite controllers, viremic controllers and progressors; the right was mainly progressors with one exception. **(B)** Venn diagram showing the numbers of candidate miRNAs filtered with different criteria. The miRNAs in the lower left and right circles were generated by significance analysis of microarrays (SAM) of participants stratified by the CD4+ T cell count (<250 cells/μL vs. >450 cells/μL) and viral load (<2000 copies/mL vs. >10000 copies/mL), respectively. The identified 15 miRNA candidates were marked in red in **(A)**. **(C)** Correlation between expression levels of miR-31 and CD4+ T cell counts in HIV-1 infected individuals (FBD, former blood donor cohort). miR-31 expression was quantified by quantitative RT-PCR, and the relationship between relative level of miR-31 and CD4+ T cell count was examined by Spearman correlation (n = 50). Red dots represent patients that eventually reached the defined endpoints. **(D)** Kaplan-Meier survival curves of FDB patients stratified by median whole blood miR-31 level during the late phase of chronic infection. **(E–G)** Kaplan-Meier survival curves of another HIV patient cohort (an acute-phase prospective men who have sex with men (MSM) cohort) stratified by plasma miR-31 levels before and after infection. Absolute CD4+ T cell count below 350 cells/μL, initiation of long-term ART, progression to AIDS and death were defined as endpoints of the study. Patients were separated into two groups stratified by the median miR-31 level in plasma collected before infection **(E)**, during acute infection phase **(F)**, during early phase of chronic infection **(G)**.

To gain insight into the roles of miRNAs during HIV-1 infection, we compared the miRNA profiles of whole blood samples from a cohort of HIV-infected former blood donors (FBDs), who donated plasma during 1995-1996 in Yuncheng city, China and contracted HIV-1 infection from common-source of contaminated blood (27, 28). The blood samples were collected during one follow-up visit in November 2010 (14-15 years post HIV infection), when the entire cohort was ART naïve; the cohort contained 4 EC (with undetectable plasma viral load), 5 VC (with plasma viral load of 50–2000 copies/mL) and 14 viremic patients (with plasma viral load >2000 copies/mL) (**Figure 1A** and **Table 1**). The miRNA profiles were clearly clustered into two major groups and all the samples from EC and VC, with only one exception, were assigned to the left group, suggesting a potential connection between miRNA expression with viral control. When patients were classified based on an immunologic definition, 6 belonged to LTNP (maintaining CD4+ T cell count >500 cells/μL over 10 years) and the rest were progressors (experiencing sustained decline in CD4+ T cell counts). The clustering of all six LTNPs in the left group (data not shown) indicated a correlation between miRNAs and disease progression. In search of miRNAs involved in both viral control and disease progression, we identified the differentially expressed miRNAs between low viral load group (<2000 copies/mL) and high viral load group (>10000 copies/mL), and those between high CD4+ T cell count group (>450 cells/μL) and low CD4+ T cell count group (<250 cells/μL). High CD4+ T cell count traditionally defines as count of 500 cells/μL or higher; we instead used 450 cells/μL as the cutoff point as 3 patients with CD4 + T cell count in the range of 477-494 cells/μL were assigned to the high CD4+ T cell count group after taking into consideration that their CD4 + T cell counts were greater than 500 cells/μL either before or after the visit. As shown in **Figure 1B**, 15 miRNAs emerged as potential miRNA candidates correlating with HIV disease from both immunologic and virological perspectives. Among the 15 miRNA candidates, 10 miRNAs, as marked in **Figure 1A**, showed expression patterns significantly correlated with CD4+ T cell counts and viral loads, according to microarray data. For validation, these miRNAs were quantified in 50 baseline samples from the FBD study (containing 5 EC, 6 VC and 39 viremic patients) by stem-loop quantitative PCR (qPCR) assays and their correlation with disease progression were subsequently assessed using the Kaplan-Meier survival analyses. To this end, we defined an absolute CD4+ T cell count of < 350 cells/μL, initiation of long-term antiretroviral therapy, or progression to AIDS and death as the endpoints. During the 5-year follow-up period, the patients in the FBD study showed divergent clinical outcomes with 33 of 50 (66.0%) reaching endpoint (**Table 1**). Among the 10 miRNAs analyzed, miR-31 has the most significant correlation with CD4+ T cell count (**Figure 1C**), and at the same time showed a correlation with disease progression as low levels of miR-31 were significantly associated with higher risk of disease progression with a Hazard Ratio (HR) of 23.57 (P = 0.0001) (**Figure 1D**).

**Table 1:** Characteristic of HIV-1 infected individuals participating in this study.

Characteristic	Training Group	Validation Group	
	FBD* (N = 23)	FBD* (N = 50)	MSM** (N = 54)
Age — yr, Median (IQR)	49 (42–53)	45 (39–51)	30 (26–40)
Male sex — no. (%)	14 (60.9)	33 (66.0)	54 (100)
Han ethnic — no. (%)	23 (100)	50 (100.0)	54 (100)
CD4^+^ T-cell count —cells/μL			
Acute infection, mean (SD)	—	—	476 (199)
Chronic infection, mean (SD)	405 (145)	440 (149)	497 (200)
Viral load — log_10_ copies/mL			
Acute infection, median (IQR)	—	—	4.28 (3.72-5.01)
Chronic infection, median (IQR)	4.20 (2.97-4.66)	4.00 (3.32-4.59)	4.14 (3.65-4.83)
genetype — no. (%)			
CRF01AE	0 (0)	0 (0)	12 (22.2)
B	0 (0)	0 (0)	9 (16.7)
B’	23 (100)	50 (100.0)	—
B/C	0 (0)	0 (0)	2 (3.7)
Missing data	0 (0)	0 (0)	31 (57.4)
CCR5Δ32 — no. (%)	0 (0)	0 (0)	—
*nef* mutation — no. (%)	0 (0)	0 (0)	—
CD38+CD8+ — %, Median (IQR)	—	27.45 (19.30-37.95)	—
Disease progressed — no. (%)	—	33 (66.0)	31 (57.4)

*FBD, Former blood donor.

**MSM, Men who have sex with men.

To further explore the role of miR-31 in HIV-1 infection, we investigated an additional HIV patient cohort, an acute-phase prospective MSM cohort comprising 54 homosexual men newly infected with HIV-1 genotypes AE, B’ or BC, which circulate prevalently in China. The first HIV-positive samples were collected individually at 12~97 days post infection (dpi); 31 of the 54 (57.4%) participants experienced a precipitous drop in the CD4+ T cell count during the 2-year observation period after HIV-1 infection, and 16 consented to start ART (**Table 1**). The subgroup with higher levels of miR-31 showed substantially higher percentage of nonprogressors than that showing low expression of miR-31. This trend was discernible when using miR-31 levels assessed before HIV infection, but the p value was insignificant (**Figure 1E**, HR = 2.99, P = 0.09). The miR-31 high expression group and low expression group showed significant difference in disease progression when using the measurement of miR-31 during acute infection phase (**Figure 1F**, HR = 2.43, P = 0.019), or the early phase of chronic infection (**Figure 1G**, HR = 2.56, P = 0.013). The relatively small size of samples collected before HIV infection is likely to account for the failure to reach significance despite a high HR value (**Figure 1E**). These data substantiated the negative correlation between miR-31 level and HIV disease progression, suggesting a potential protective role of miR-31 in HIV disease.

### miR-31 Regulates CD4+ T Cell State

**Figure 2:**
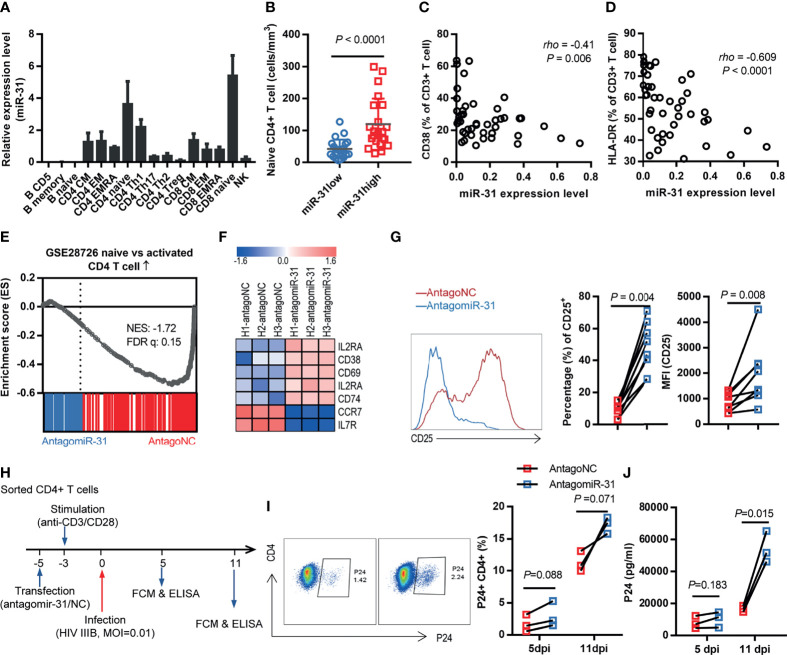
Loss of miR-31 triggers CD4+ T cell activation. **(A)** miR-31 levels in different immune cell subtypes. Data were obtained from a miRNA RTqPCR data from Rossi *et al*’s work (see the text for reference). **(B)** Comparison of absolute naïve CD4+ T cell counts in blood of HIV-1 infected individuals (FBD, n = 50) stratified by miR-31 expression. **(C, D)** Correlation between miR-31 levels and frequencies of CD38+ T cells **(C)** or HLA-DR+ T cells in blood of HIV infected individuals (FBD, n=44) **(E)** Gene set enrichment analysis (GSEA) of “naïve” signature in antagomiR-31- *versus* antagoNC- treated naïve CD4+ T cells. NES, normalized enrichment score. **(F)** Heatmap of representative genes associated with activation *versus* naïve state of T cells. Shown is log2 fold changes of gene expression in antagomiR-31-treated naïve CD4+ T cells relative to that in antagoNC-treated cells (n=3). **(G)** Effects of antagomiR-31 treatment on CD25 expression of naïve CD4+ T cells, assessed by frequency of CD25+ cells and median fluorescent intensity (MFI) of CD25. Average fold changes were 4.45 and 2.25, respectively (n = 8). Blue, antagomir-31 treated group; red, antagoNC-treated group. Representative FACS data for CD25 were shown in the left panel. **(H)** Schema of the *in vitro* assay used for examining the role of miR-31 in HIV-1 infection. CD4+ T cells were sorted, followed by transfection with antagomiR-31 or antagoNC. After 48 hours, cells were stimulated with a mix of anti-CD3 and anti-CD28 antibodies and infected with HIV-1 IIIB 5 days later. **(I, J)** Cells and supernatants were collected on days 5 and 11 post infection and respectively subjected to flow cytometry for determination of P24-expressing CD4+ T cells **(I)** and ELISA for quantification of released P24 proteins **(J)** (n = 3).

To explore the mechanism by which miR-31 slows HIV-1 disease progression, we examined the expression of miR-31 in immune cell types in peripheral blood by analyzing a miRNA RTqPCR data from Rossi *et al*’s work (29). The analysis indicated that miR-31 was expressed at a much higher level in naïve T cell subsets than in non-naïve T cell subsets (**Figure 2A**). Characterization of blood samples from FDB cohort evidenced the link between miR-31 and naïve state of CD4+ T cells. When split into two halves according to the expression level of miR-31, the abundance of naïve CD4+ T cells was 2.8-fold higher in the group with high miR-31 expression, denoted as miR-31high, relative to the group showing relatively lower miR-31 level, namely miR-31low group (**Figure 2B**; P < 0.0001). We also observed a negative correlation between miR-31 expression with levels of two T cell activation markers, CD38 and HLA-DR (**Figures 2C**, **D**). Following this, we interrogated the microarray data sampling whole-blood RNA of 8 individuals from each of the miR-31high and miR-31low groups. Among the many differentially expressed genes between miR-31high and miR-31low groups, gene ontology analyses showed enrichment in the categories of cell death, cell cycle regulation, and T Cell activation (**Supplementary Figure 1**), indicating that miR-31 might regulate T cell activity through multiple mechanisms.

To corroborate these observations *in vitro*, we sorted naïve CD4+ T cells from healthy donors and subsequently treated them with either antagomiR-31 or antagomir mismatch control (antagoNC), followed by mRNA isolation 48 hrs later for RNA-seq analysis. The knockdown efficiency was >90% as determined by quantitative RT-PCR (**Supplementary Figure 2**). As compared to antagoNC-treated cells, the antagomiR-31-treated cells showed a transcriptional profile tilted more toward activation-like status, exemplified by consistent upregulation of activation markers, including IL2RA (CD25), CD38, CD69, IL2RB, and CD74, and downregulation of CCR7 and IL7R (CD127), which characterize no or low effector functions (**Figures 2E**, **F**). For validation, we assessed the membrane expression of CD25, CD38, CD69, CCR7 and CD127 by flow cytometry. Consistent with the RNA-seq data, the CD25+ subset was significantly expanded upon antagomiR-31 treatment relative to antagoNC treatment (**Figure 2G**), while a weak but discernible elevation was observed in CD38 and CD69 expression (**Supplementary Figures 3A, B**). Contrastingly, the expression of two stemness markers, CCR7 and CD127, were downregulated upon miR-31 inhibition (**Supplementary Figures 3C, D****)**. Thus, miR-31 inhibition promoted the acquisition of an activation-like status in naïve CD4+ T cells, substantiating a connection between miR-31 level with T cell homeostasis.

Activated, proliferating CD4+ T cells are highly susceptible to infection and support efficient HIV-1 replication (30). If our notion that the level of miR-31 gates the activation status of CD4+ T cells, we would predict that impairing miR-31 expression would render enhanced susceptibility of CD4+ T cells to HIV-1 replication, recapitulating the relationship we observed with HIV patients (**Supplementary Figure 4**). To this end, we isolated CD4+ T cells from healthy donors and then transfected them with antagomiR-31 or antagoNC, followed by 72-hr stimulation with anti-CD3/CD28 antibodies before infection with HIV-1 at a multiplicity of infection (MOI) of 0.01 (**Figure 2H**). As detected by flow cytometry, the percentage of cells expressing HIV P24 antigen increased over time for both antagomiR-31- and antagoNC-treated cells, however the former showed an approximately 3-fold elevation relative to the latter at 11 days post infection (**Figure 2I**). The difference in infection rate was corroborated by significantly increased level of p24 protein in the supernatants of antagomiR-31-treated cells (**Figure 2J**). This *in vitro* result strengthened our postulation that miR-31 is a major determinant of susceptibility of CD4+ T cells to HIV infection *via* governing their activation status.

### Inhibition of miR-31 Leads to a Th1 Biased Response

**Figure 3:**
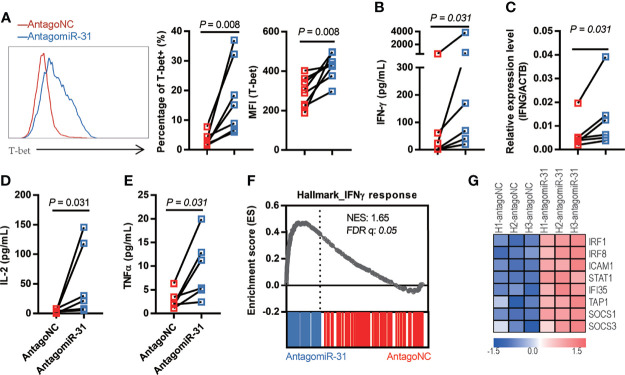
Loss of miR-31 drives the Th1-biased response of naïve CD4+ T cells. **(A)** Effect of antagomiR-31 *versus* antagoNC treatment on the T-bet expression of naïve CD4+ T cells from healthy donors. Flow cytometry analysis was performed at 48hrs post treatment, shown are representative FACS plot (left), percentages of T-bet+ cells (middle), and MFI of T-bet (right). The relative fold changes in T-bet+ cell percentage and MFI of T-bet were 5.46 and 1.45 respectively (n = 8). **(B–E)** Supernatants from antagomiR-31- and antagoNC-treated naïve CD4+ T cells (n = 6) were collected at 48hrs post treatment for determination of IFN-γ **(B)**, fold change = 7.81), IL-2 **(D)** fold change = 14.68) and TNF **(E)** fold change = 3.42) using cytometric bead array (n = 6). The cells were also harvested for determination of IFN-γ mRNA levels by quantitative RT-PCR [**(C)**, fold change= 2.08]. **(F)** GSEA plot of enrichment in IFN-γ response gene signature in antagomiR-31-treated naïve CD4+ T cells. **(G)** Heatmap of the representative core enrichment genes involved in IFN-γ response.

Strong TCR signaling favors the development of Th1 cells (31), and a driving force of this process is provided by the action of transcription factor T-bet, which undergoes transient upregulation in naïve T cells after TCR stimulation (32, 33). Given the above demonstration of connection of miR-31 to T cell activation status, we sought to characterize whether inhibition of miR-31 leads to the induction of a Th1-biased response. Thus, we sorted naïve CD4+ T cells from healthy donors and treated these cells with antagomiR-31- and antagoNC same as above. As analyzed by flow cytometry, the expression of T-bet was significantly higher in antagomiR-31-treated cells than antagoNC-treated cells, with 5.4-fold (P = 0.0078) and 1.4-fold (P = 0.0078) increase in percentage of T-bet-positive cells and mean fluorescence intensity (MFI) respectively (**Figure 3A**). The production of IFN-γ, both in protein level in the supernatant assessed by Cytometric Bead Array (CBA) and in cellular mRNA level determined by quantitative RT-PCR, was concomitantly increased by antagomiR-31 treatment (**Figures 3B**, **C**). Two other Th1 cytokines, IL-2 and TNF, were also notably upregulated (**Figures 3D, E****)**. The upregulation of IFN-γ signaling was further revealed by gene set enrichment analyses (GSEA), within which antagomiR-31-treated naïve CD4+ cells, in comparison to those receiving antagoNC, showed significant enrichment of IFN-γ pathway (**Figure 3F**), exemplified by a few well characterized IFN stimulated genes (ISGs) including IRF1, IRF8, ICAM1, STAT1, IFI35, TAP1, SOCS1, and SOCS3 (**Figure 3G**). Taken together, these data suggested that a major impact of downregulation of miR-31 on naïve CD4+ T cells is enhancement of T-bet signaling and consequently induction of a Th1-biased response, which might account for the enhanced susceptibility to HIV.

### miR-31 Suppresses STAT1-T-Bet Pathway by Directly Targeting STAT1

**Figure 4:**
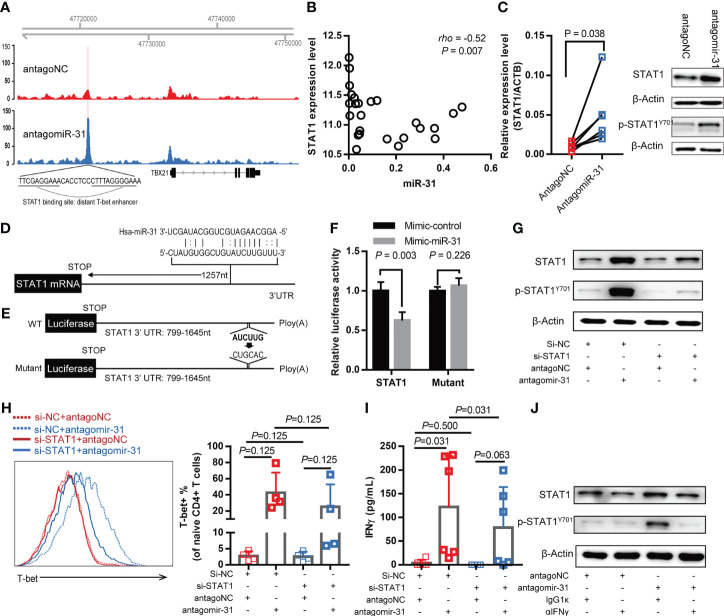
miR-31 negatively regulates the IFN-γ-STAT1-T-bet axis by directly targeting STAT1. **(A)** Genome browser view of accessible chromatin regions in the *TBX21* locus, which encodes T-bet, in antagomiR-31-treated cells *versus* antagoNC-treated naïve CD4+ T cells. The differential peak was located at a distant enhancer containing a STAT1 binding site. Results are representative of ATAC-seq data obtained from two different donors. **(B)** Correlation between miR-31 level and STAT1 mRNA level in blood samples of HIV infected individuals (n = 25), measured by microarray and quantitative RT-PCR respectively. **(C)** Comparison of STAT1 mRNA and protein levels between antagomiR-31- and antagoNC-transfected naïve CD4+ T cells. mRNA levels of STAT1 were measured by quantitative RT-PCR and normalized to the level of ACTB (left panel, fold change = 5.10, n=6). Total STAT1, phosphor-STAT1 and internal control β‐actin were detected by western blotting, the representative immunoblot from 4 healthy donors was shown in the right panel. **(D)** Schematic illustration of putative miR-31 target site in the STAT1 3’ UTR. **(E)** Luciferase reporter constructs to verify the STAT1 targeting by miR-31. The nucleotide alterations intended to abolish the putative miR-31 target site was indicated. **(F)** Activities of luciferase reporter construct in 293T cells transfected with control mimic or miR-31 mimic. Data are presented as mean ± SD (n = 4). **(G–I)** Naïve CD4+ T cells were co-transfected with indicated 5’-cholesterol modified siRNA and antagomir-31 or antagoNC. After 48 hrs, cells were harvested and subjected to western blotting for detection of total STAT1 and phospho-STAT1 (n=3) **(G)** and flow cytometry for assessment of T-bet-expressing cells **(H)**. The supernatants were also collected for IFN-γ measurement using CBA **(I)**. The T-bet and IFN-γ measurements were expressed as mean ± SD (n=6). **(J)** IFN-γ dependency of antagomir-31-mediated STAT1 activation. Antagomir-31 or antagoNC-transfected naïve CD4+ T cells were exposed to IFN-γ neutralizing antibody or isotype control antibody for 48 hrs, whole cell lysates were prepared for detection of total STAT1, phospho-STAT1, and β‐actin using western blotting (n=3).

To explore the mechanism by which miR-31 regulate T-bet signaling, we performed ATAC-Seq to compare the chromatin accessibility landscape of antagomiR-31-treated naïve CD4+ T cells to that of antagoNC-treated control cells. Among differentially accessible chromatin regions (peaks) displayed by antagomiR-31-treated cells, an informative one was mapped to ~11.7 kb upstream of TBX21 gene transcription start site (**Figure 4A**), corresponding to a previously identified distant enhancer element featuring STAT1 binding motifs (34). This data, which can be explained by recruitment of activated STAT1 to the mapped distant enhancer element, was in line with that *STAT1* was among the most significant differentially expressed genes associated with antagomiR-31-treatment (**Figure 3G**). Linking these observations to the fact that STAT1 is required for the upregulation of T-bet during naïve CD4+ T cell activation (35), we thus hypothesized that miR-31 might control the activation status of CD4+ T cell *via* the STAT1-T-bet-IFN-γ circuit.

Following this, we first analyzed the relationship between STAT1 and miR-31 levels in whole blood samples of HIV-1-infected individuals. A significant negative correlation was detected, providing the first evidence supporting our hypothesis (**Figure 4B**). We also assessed the correlation between miR-31 and specific phenotypic markers, and found that miR-31 was negatively correlated with two stemness-related markers, CCR7 and IL7R (**Supplementary Figure 5**). These correlation assessments, combined with the positive correlation between miR-31 and activation marker CD38 and HLA-DR as shown in **Figures 2C** **, D**, substantiated the notion that the naïve state of CD4+ T cells is preserved at least in part by miR-31. The regulation of STAT1 by miR-31 was further validated in antagomiR-31-treated naïve CD4+ T cells, which exhibited increased STAT1 mRNA and protein abundance as well as upregulated phospho-STAT1 level when compared to antagoNC-treated control cells (**Figure 4C**). To investigate whether miR-31 directly regulate STAT1, we used bioinformatics to predict the potential miR-31 targeting of STAT1. One algorithm provided by miRWalk was able to identify a putative miR-31 target site in the 3’ untranslated region (UTR) of STAT1 mRNA (**Figure 4D**). Subsequently, we made two luciferase reporter constructs containing either wild-type STAT1 3’ UTR or its mutant with the predicted miR-31 target site being eliminated for evaluation of the effect of miR-31 co-expression (**Figure 4E**). A significant miR-31-mediated repression of luciferase reporter gene was observed with the construct containing wild type 3’ STAT1 UTR but was rescued by mutation of the predicted miR-31 target site (**Figure 4F**). Together, these experiments proved that STAT1 is a cognate target of miR-31.

**Figure 5:**
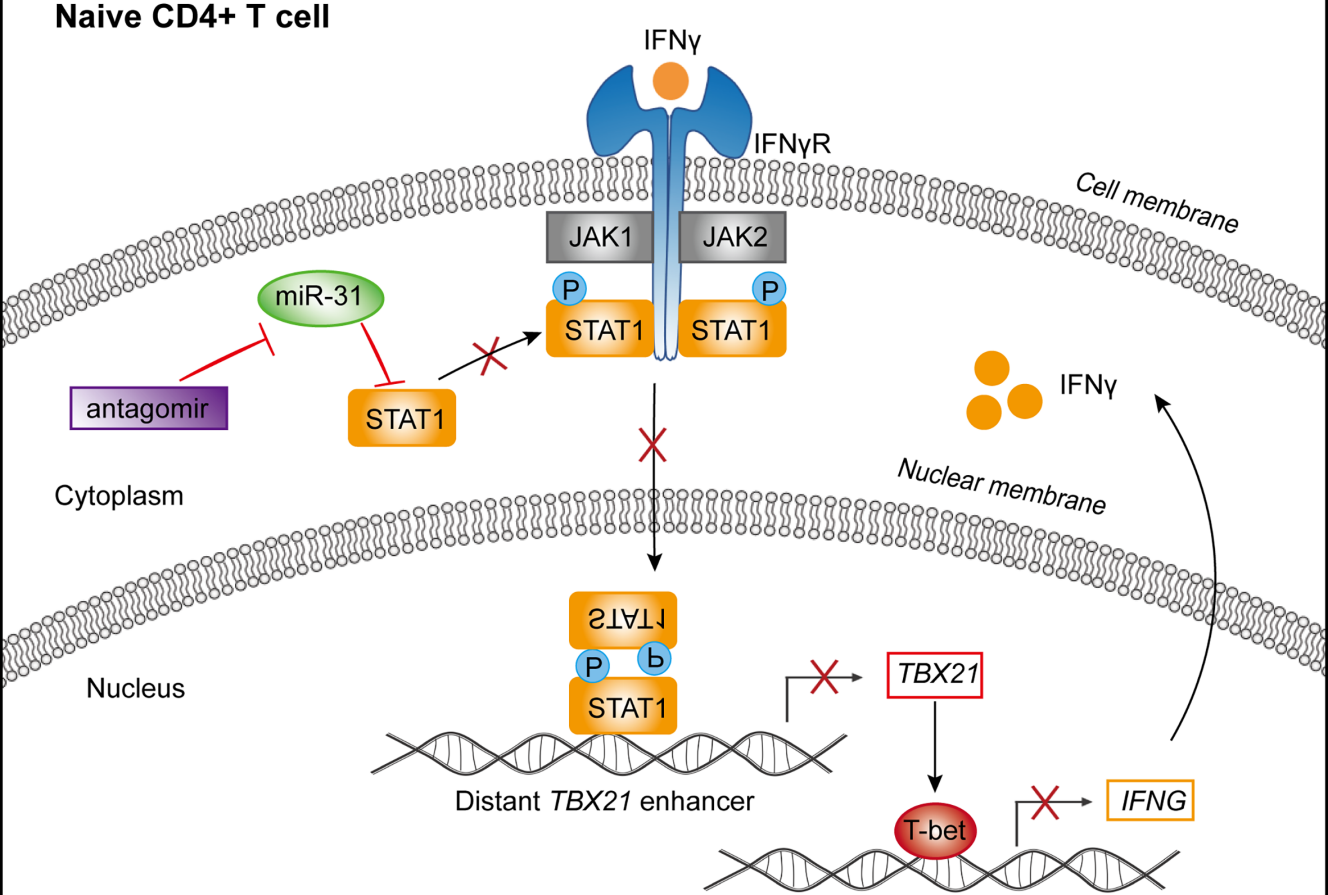
Schematic representation of the mechanism by which miR-31 regulates the homeostasis of CD4+ T cells. miR-31 downregulates the STAT1-T-bet-IFN-γ feedback loop *via* directly targeting STAT1, thus serving as a gatekeeper of CD4+ T cell activation.

Next, we evaluated the contribution of STAT1 downregulation to the activity of miR-31 in modulating CD4+ naïve T cells. To this end, we employed 5’-cholesterol modified siRNA to inhibit STAT1. Treatment with STAT1-specific siRNA (si-STAT1) successfully reduced, although not abolished, the antagomiR-31-induced upregulation of STAT1 and phospho-STAT1 (**Figure 4G**). As assessed by flow cytometry, the antagomiR-31-induced T-bet upregulation was substantially prevented by si-STAT1 treatment (**Figure 4H**), so was the elevated IFN-γ protein level in the supernatant as determined by CBA (**Figure 4I**). Finally, we determined the requirement of IFN-γ signaling for the antagomiR-31-mediated activation of STAT1. In the presence of an IFN-γ neutralizing antibody, the increment of total STAT1 and phospho-STAT1 induced by antagomiR-31 were both largely blocked (**Figure 4J**). We thus concluded that the regulatory role of miR-31 in the homeostasis of naïve CD4+ T cells is at least in part attributable to its gating action on the STAT1/T-bet/IFN-γ positive feedback loop *via* directly targeting STAT1 (**Figure 5**).

## Discussion

Up to now, a few studies investigated the utility of miRNAs as biomarkers of clinical outcome in patients with HIV infection (20, 21, 36). Here, based on a 5-year follow up study on a unique cohort of HIV-infected former blood donors, we correlated miRNA profiling of blood sample with disease progression and clinical parameters. Consequently, we uncovered miR-31 as the most distinguished protective miRNA in our miRNA panel. Following characterization of miR-31 in human T cells revealed that it is preferentially expressed in naïve subsets and its downregulation promotes polarization of isolated human naïve CD4+ T cells into a Th1 phenotype. We subsequently identified the mechanism by which miR-31 modulates the activation status of CD4+ T cells, that is, it directly targets 3’UTR of STAT1 and thereby serves as a molecular brake on the IFN-γ-Stat1-T-bet positive feedback loop, which is a major driving force for T cell activation.

We presented several lines of evidences supporting the linkage between miR-31 and HIV disease progression. MiR-31 expression was found to be positively correlated with CD4+ T cell count while reversely correlated to viral load. Patients exhibiting high miR-31 expression in blood sample appeared to experience a slower disease progression as compared to those with relatively lower blood level of miR-31, whereas the opposite was observed for CD4+ T cell counts. Importantly, this marked difference was also observed for acute HIV-1 infections. These data are in line with previous studies suggesting the potential association of miR-31 with HIV infection. Interestingly, besides miR-31, there were no other miRNAs emerging in our study as a sole independent biomarker of the HIV disease progression/phenotypes, including those that were implicated in previous studies as potential associated factors for HIV infections. A plausible explanation for such discrepancy is that these miRNAs might still relate to HIV infection, however, with a less prominent role compared to miR-31, they can thus only be revealed in certain contexts, such as within a special patient cohort. In this respect, it should be noted that, although the chronic infection cohort in our study was predominantly infected by Thailand B clade, the studied acute infection cohort covered multiple subtypes of HIV-1 circulating in China (21), including HIV-1 B, C, CRF_07BC, and CRF_01AE. Therefore, the observed effect of miR-31 on HIV-1 infection is unlikely to be confined to patients contracting a particular HIV-1 clade.

The new identification of miR-31 as a gatekeeper of naivety of CD4+ T cells deviated significantly from current knowledge of T cell-regulating function of miR-31, which is largely derived from mouse studies. Mouse miR-31 (mmu-miR-31) undergoes substantial transcriptional induction following T cell activation and was characterized as a facilitator of CD8+ T cell dysfunction during chronic stage of infection. Mechanistically, mmu-miR-31 renders T cells more sensitive to type I interferons, thereby downregulating the effector T cell function while upregulating the expression of multiple inhibitory molecules to cause T cell exhaustion (37). In sharp contrast, our study showed that human miR-31 (hsa-miR-31) is predominantly expressed in naïve subsets of T cells. This finding is in line with previous studies, which also showed rapid downregulation of hsa-miR-31 upon activation of human naïve T cells (38, 39). The connection of hsa-miR-31 to homeostasis of naïve CD4 T cells was built on two observations: Firstly, our RNA-seq data revealed that repression of hsa-miR-31 in naïve CD4+ T cells produced a mRNA profile indicative of acquisition of effector cell phenotype, marked by reduced expression of naïve-related genes such as IL7R and CCR7 and significant induction of activation-related genes including IL2RA/CD25, IL2, CD38, CD69. Secondly, naïve CD4+ T cells are resistant to productive HIV infection due to their quiescent state (40), however, their number dramatically decreases as HIV disease progresses. Consequently, we showed that antagonizing hsa-miR-31 increased the *in vitro* susceptibility of T cells to HIV infection, strengthening the view of hsa-miR-31 being a key protective factor during the HIV pathogenesis as a product of its function in maintaining a naïve phenotype.

Our characterization of the effect of change in hsa-miR-31 level on CD4+ T cell activity provided further insights into the crucial role of hsa-miR-31 in governing T cell activation. The analysis of samples from HIV patients revealed that low hsa-miR-31 expression was associated with upregulation of CD38 and HLA-DR, two surface markers linked to T cell activation. This was corroborated by our *in vitro* study showing inhibition of hsa-miR-31 in naïve CD4+ T cells inducing characteristic Th1 response, including release of proinflammatory cytokines (IL-2, IFN-γ and TNF-α) and heightened expression of T-bet transcription factors, even under non-stimulating conditions. These findings are consistent with the notion that hsa-miR-31 acts as a molecular rheostat for T cell activation. Thus, hsa-miR-31 not only grant the activation license but also may determine the order of CD4+ T cell subsets for differentiation after naïve CD4+ T cells are activated.

The identification of STAT1 as a novel target of hsa-miR-31 elucidate a plausible mechanism accounting for the rheostat activity of hsa-miR-31. T-bet expression during T cell activation is known to be regulated by IFN-γ signaling in STAT1-dependent manner. At the other end, T-bet is essential for Th1 cell program and is required for optimal production of IFN-γ through transcriptional activation. Thus, IFN-γ-STAT1-T-bet axis functions as a positive feedback loop allowing rapid and potent T cell activation. High expression of hsa-miR-31 keeps this loop in check in naïve CD4+ T cells as hsa-miR-31 downregulates STAT1 by directly targeting its 3’ UTR. Such checking would be relieved upon reduction in hsa-miR-31 expression, which might be triggered when the TCR signaling exceeds a certain threshold, thus promoting differentiation into effector cells. It is tantalizing to speculate that the level of hsa-miR-31 is also governed by IFN-γ-STAT1-T-bet axis, thus these molecules make a four-component circuit capable of robust fine-tuning of CD4+ T cell fate. Dysregulation of this circuit like that with lowered hsa-miR-31 expression would skew the balance toward the end of activation, which is translated into increased HIV susceptibility and accelerated disease progression in case of human HIV infection, as Th1 cells are highly sensitive to activation-induced T cell death (AICD) and are lost more rapidly than other Th subsets (41). Along the same line, higher levels of STAT1 in CD4+ T cells were reported to be associated with lower CD4+ T cell counts in HIV patients (42). Intriguingly, although mmu-miR-31 regulates the response of CD8+ T cells to type I interferon, it has little or no effect on murine STAT1 expression. This can be explained by that the hsa-miR-31 targeting site in the 3’ UTR of human STAT1 is not conserved in murine STAT1. Moreover, mmu-miR-31 is upregulated in murine Th1 cells upon periodic TCR activation (43), which is in sharp contrast to preferential expression of hsa-miR-31 in human naïve CD4+ T cells, as shown here and in previous reports (38, 39). Why an important regulator like miR-31 employs different schemes to regulate T cell response in different species is an interesting question for future investigation.

In summary, our data established hsa-miR-31 as a critical biomarker of HIV disease progression and more importantly unraveled its identity as a key regulator of homeostasis of naïve CD4+ T cells, highlighting the connection between CD4+ T cell activation and HIV susceptibility. One important implication of this study is that delivery of hsa-miR-31 into CD4+ T cells might present a novel approach to combine with ART for treatment of HIV patients as the combination will bestow a synergistic effect in immune reconstitution.

## Data Availability Statement

The datasets presented in this study can be found in online repositories. The names of the repository/repositories and accession number(s) can be found below: GSE56837, GSE36557 and PRJNA756859 (https://www.ncbi.nlm.nih.gov/).

## Ethics Statement

The studies involving human participants were reviewed and approved by the Ethics Committee of Shanghai Public Health Clinical Center. The patients/participants provided their written informed consent to participate in this study.

## Author Contributions

JX, XZ, JL, HW, and CZ designed the experiments. JX and LZ interpreted the results and wrote the paper. LZ performed most of the experiments. CQ, LD, LXZ, MF, YY, AZ, JH, and WF contributed to HIV-1 cohorts sample collection and processing and data analysis. CLQ performed CD4+ T cell count experiments. YWang contributed to viral load detection. YWan performed RNA-seq experiments. All authors contributed to the article and approved the submitted version.

## Funding

This study was supported by Natural Science Foundation of China (Grant No. 82001679), Shanghai Sailing Program (Grant No. 20YF1441500), and Chinese National Grand Program on Key Infectious Disease Control (Grant No. 2017ZX10202102-001).

## Conflict of Interest

The authors declare that the research was conducted in the absence of any commercial or financial relationships that could be construed as a potential conflict of interest.

## Publisher’s Note

All claims expressed in this article are solely those of the authors and do not necessarily represent those of their affiliated organizations, or those of the publisher, the editors and the reviewers. Any product that may be evaluated in this article, or claim that may be made by its manufacturer, is not guaranteed or endorsed by the publisher.
