# Supplementary material for: Hsa-miR-31 Governs T-Cell Homeostasis in HIV Protection via IFN-γ-Stat1-T-Bet Axis
Source: Front Immunol. 2021 Nov 5;12:771279. doi: 10.3389/fimmu.2021.771279 (PMC8602903; doi:10.3389/fimmu.2021.771279)
Supplement: Supplementary file 1 [file DataSheet_1.docx]

**Supplemental Figures
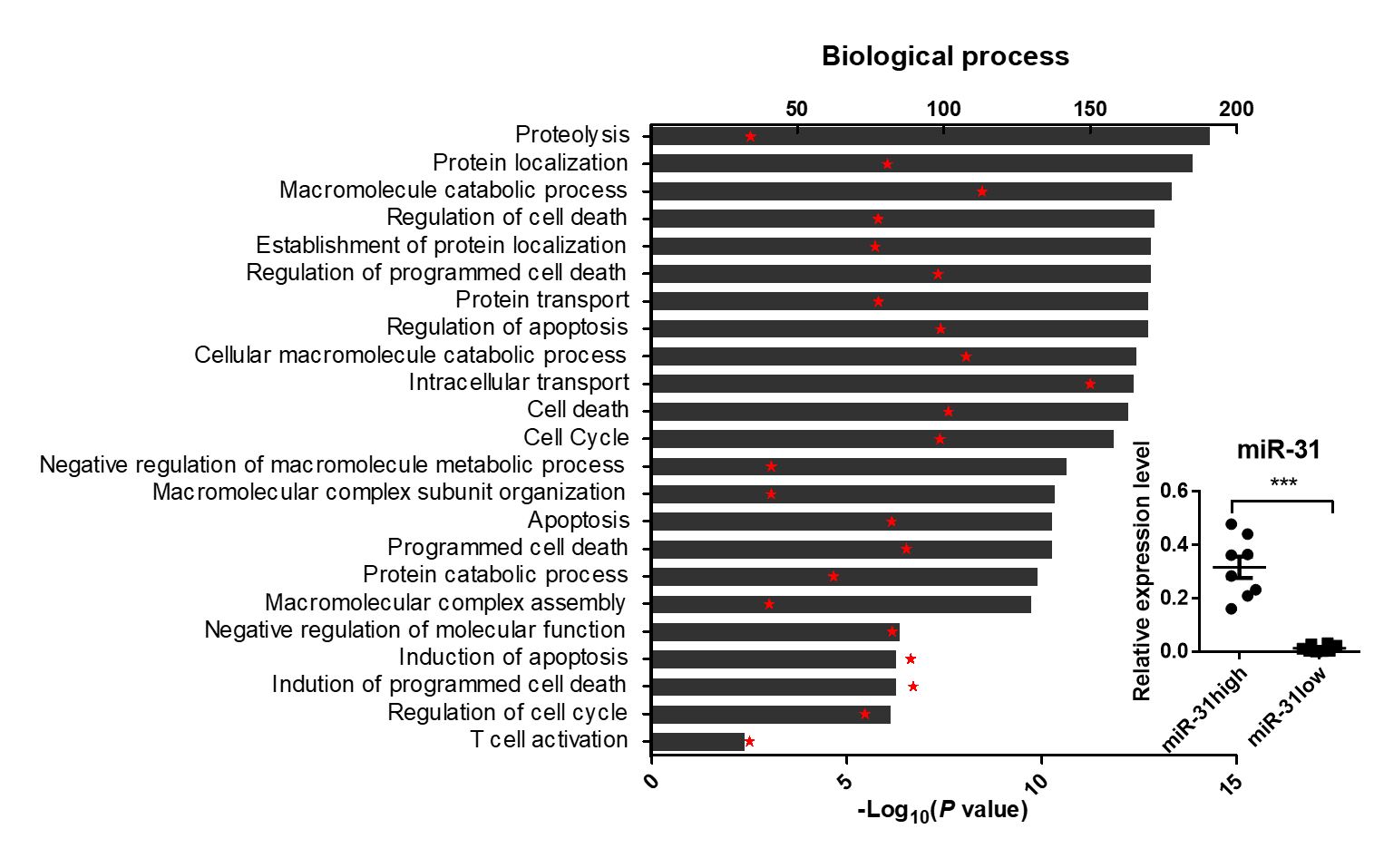
**

**Fig. S1. Lower miR-31 level is associated with higher expression of genes involved in T cell activation and proliferation, cell cycling and cell death *in vivo*.** Blood miR-31 levels were quantified for 30 HIV-1 infected individuals, 8 individuals with highest miR-31 expression (miR-31high) and 8 individuals with the lowest miR-31 expression (miR-31low) were selected as miR-31high group and miR-3low group (P < 0.001 for the difference in miR-31 levels between the two groups, fold change = 23.47). Shown are GO analyses of differentially expressed genes between the two groups. “Red star” indicates log10 (P), the height of “black bar” represents gene numbers enriched in each GO category.


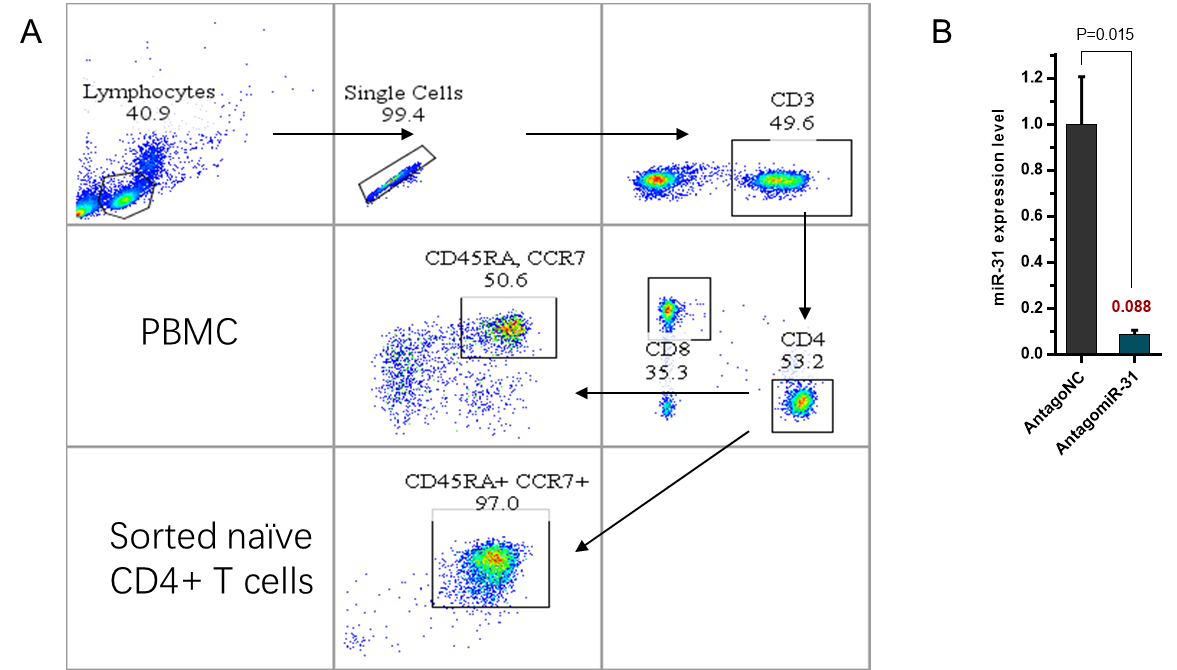


**Fig. S2. MiR-31 knockdown efficiency in naïve CD4 T cell**. (A) Representative plots to illustrate gating strategy used to identify naïve CD4 T cells. (B) Knockdown efficiency of miR-31 in purified naïve CD4 T cells 48 hrs after antagomir-31 transfection, assessed by quantitative RT-PCR.


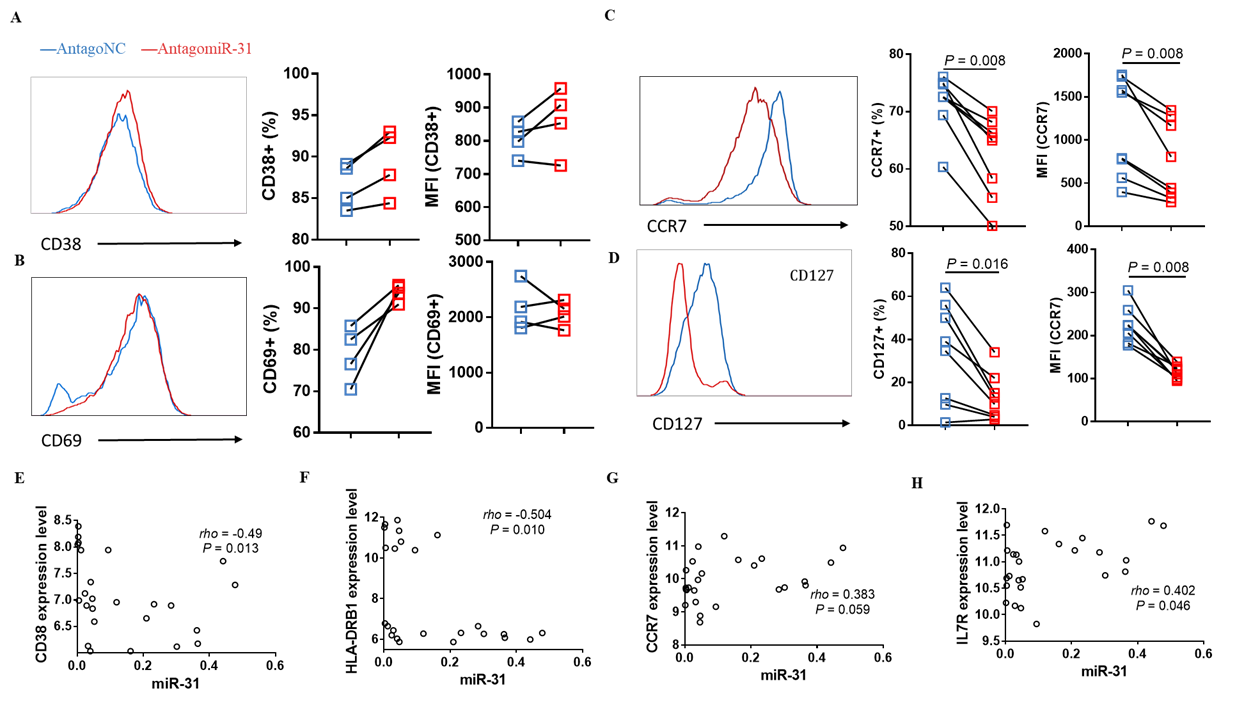


**Fig. S3.** **Effects of antagomiR-31 treatment on surface marker expression of naïve CD4+ T cells.** The assayed markers included CD38 (A), CD69 (B), CCR7 (C) and CD127 (D). Blue, antagomir-31 transfected group; red, antagoNC transfected group. Shown from left to right were representative FACS plot, frequency of positive cells, and median fluorescent intensity (MFI).

**Fig. S4. MiR-31 level was negatively correlated with viral load**. Relative blood levels of miR-31 were plot against plasma viral loads for HIV-1-infected individuals (n=50) (P < 0.001, Spearman rho = -0.482).


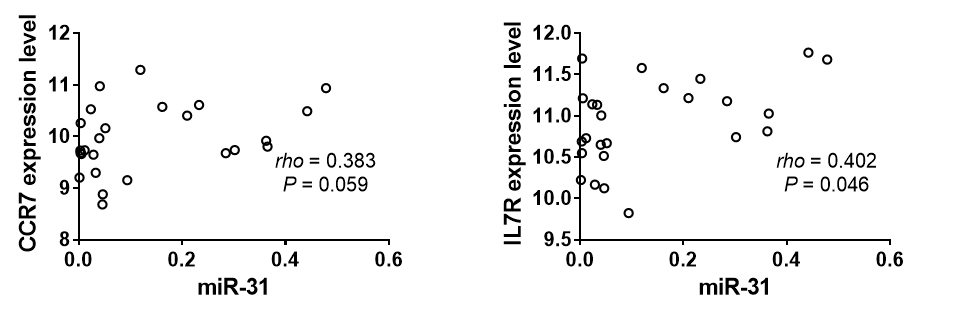


**Fig. S5**. **Correlation of miR-31 to CCR7 and IL7R.** The measurements were performed with blood samples of HIV infected individuals (n = 25), with levels of miR-31 and CCR7/IL7R being assessed by microarray and quantitative RT-PCR, respectively.
